# Supplementary material for: Patterns of Intron Gain and Loss in Fungi
Source: PLoS Biol. 2004 Nov 30;2(12):e422. doi: 10.1371/journal.pbio.0020422 (PMC532390; doi:10.1371/journal.pbio.0020422)
Supplement: Table S1 — Also available at http://genes.mit.edu/NielsenEtAl/. (4.3 MB ZIP). [file pbio.0020422.st001.zip › NielsenEtAl/html/150.html]

AN9072.1.NCU00889.1.MG01179.1.FG00808.1


```
 CLUSTAL W (1.82) Multiple Sequence Alignments - Introns Inserted


Sequence 1: NCU00889.1	364 aa
Sequence 2: FG00808.1	357 aa
Sequence 3: MG01179.1	342 aa
Sequence 4: AN9072.1	358 aa
Alignment Length: 416 aa
Number Identitical Residues: 159 aa
Alignment Score (without introns) 6950


MG01179.1 	MARPKRSNSAASEESTATVRDQ0ELGSMYDYLAKIILLGPSGTGK2ASPATTRSCLLHRF
NCU00889.1	MTRPRRS-SATSEESAGTGREQ0ELGSMYDYLAKIILLGPSGSGK2-------SCLLHRF
FG00808.1 	MTRPRRH-SGASEESSGTAREQ0ELGSMYDYLAKIILLGPSGTGK2-------SCLLHRF
AN9072.1  	MVRHRGH-SNASSASTLPDRNQ0ELESMYDYLAKVILLGPSGAGK2LSRAGIASSLIH--
          	*.* :   * :*. *: . *:* ** ********:*******:**  : :   *.*:*  

MG01179.1 	VKNEWRVLSSQTIGVEFASKIIKVGTGARRKRIKL------------------QLWDTAG
NCU00889.1	VKSEWRVLSSQTIGVEFASKIIKVGTGARRKRIKL------------------QLWDTAG
FG00808.1 	VKNEWRVLSSQTIGVEFATKIIKVGTGSRRKRIKLQVHHSLSRNNREWDIDVTQLWDTAG
AN9072.1  	LAPVGRVLSSQTIGVEFSSRIVKLGTGPRRTRIKL------------------QLWDTAG
          	:    ************:::*:*:***.**.****                  *******

MG01179.1 	TERFRSVSRSYYRGAAGAVLVYDVTSHSTFDALPAFLNDARALASPNLTLLLAGNKVDLA
NCU00889.1	TERFRSVSRSYYRGAAGAVLCYDITSHNSFSNLQPFLNDARALASPNLSLILVGNKLDLA
FG00808.1 	TERFRSVSRSYYRGAAGAILVYDLTSHASFRNLQPFLNDARALASPNLSLMLVGNKVDLT
AN9072.1  	TERFRSVSRSYYRGAAGAILIYDVASYASFNSLPTFLMDARALTSPYLTVILAGNKTDLT
          	******************:* **::*: :*  * .** *****:** *:::*.*** **:

MG01179.1 	-------QDDLMGFGDDADGAPQSSGAS---QLSSSFPAGT------------SVASSSS
NCU00889.1	GGGHPGDEEDLLG-PLPPPTPSSTSAHSIPYTISSVHTGSAGVSVAGTPLQAGSYASSTT
FG00808.1 	--------DTLVDTSLPPPTPNSVTSNS---TLTSGMLGGGGS----------SYTSTAT
AN9072.1  	-------QDDYHEDGMRRPITPSSTSSP-----QSSLP----------------YDSTAG
          	       .:           . . :. .      *                     *:: 

MG01179.1 	AWGGGGAGLG--------TQMRASTAPDGREVGAAEASRWASAAGIPVAVEVSALSGEGV
NCU00889.1	STIRPGDSTSTSYGYGLGSQLQATVAPDGREVSAVDASRWASMVNVPVTMEVSAFSGEGV
FG00808.1 	TRDR-GASIS------AGNQLRATIAPEGREVTRAESSRWASTAGISVVTEASAFNGEGV
AN9072.1  	SFRSSNFGTA--------TRMTATYASHGREVSMEEASQWAARSNIPAVVEVSALTGDGV
          	:    . . .        .:: *: *..****   ::*:**:  .:... *.**:.*:**

MG01179.1 	DDIFGRLARQILTKIELGEIDPDDPMSGIQYGDAGVSWG-PGSDVASIKSSMTGDD----
NCU00889.1	DEVFGRLARMILTKIELGEIDPDDPMSGIQYGDYVGGWSGGASDGASIKSSMTGD-----
FG00808.1 	DEIFERLARIILTKIELGEIDPDDPASGIQYGD-SGGWN-TASDGGSIKSSMTGATVDDV
AN9072.1  	EELFQRLARIILTKIELGEIDPDDPQSGIQYGD-GSPYGHGTSDASSIKSQMTIEDNAVQ
          	:::* **** *************** *******    :.   ** .****.**       

MG01179.1 	GGGTRRRRGRGRKSVMALREWEEVFT~LNR--------------GRRGANCC-------
NCU00889.1	EQGTRNRRRKPRRGVQGLREWEEVFT~LTSG-------------RRRNNGCC-------
FG00808.1 	HNGLRRRRKKNRTQNWGLREWEEVFT~LSS--------------RRRGGGCC-------
AN9072.1  	LHRRNTRRRGGSNWRASMNEWEDVFR0AVADAPAYFSLYSDILAVTRPASVSRFHTPYF
          	    . **        .:.***:**     .:.:  :  :.  :  *  . .   :.
```
